# Psychosocial Correlates of Childhood Body Mass Index: Racial and Ethnic Differences

**DOI:** 10.31586/gjcd.2025.1180

**Published:** 2025-02-13

**Authors:** Shervin Assari, Hossein Zare

**Affiliations:** 1 Department of Internal Medicine, Charles R. Drew University of Medicine and Science, Los Angeles, CA, United States; 2 Department of Family Medicine, Charles R. Drew University of Medicine and Science, Los Angeles, CA, United States; 3 Department of Urban Public Health, Charles R. Drew University of Medicine and Science, Los Angeles, CA, United States; 4 Marginalization-Related Diminished Returns (MDRs) Center, Los Angeles, CA, United States; 5 Department of Health Policy and Management, Johns Hopkins Bloomberg School of Public Health, Baltimore, MD, United States; 6 School of Business, University of Maryland Global Campus (UMGC), Adelphi, MD, United States

**Keywords:** Body Mass Index, Socioeconomic Status, Family SES, Neighborhood SES, Inhibitory Control, Impulsivity, Racial Disparities, Ethnic Disparities, Health Disparities, Structural Inequities, Behavioral Traits

## Abstract

**Objective:**

To examine racial/ethnic differences in the associations of family socioeconomic status (SES), neighborhood SES, and inhibitory control with body mass index (BMI) in 9–10-year-old children using data from the Adolescent Brain Cognitive Development (ABCD) study.

**Methods:**

This cross-sectional study included a diverse sample of children aged 9–10 years, representing non-Latino White, Black, Latino, Asian, and Other racial/ethnic groups. BMI was the primary outcome. Key predictors were family SES, neighborhood SES, and inhibitory control. Multivariable regression models were stratified by race/ethnicity to identify group-specific associations.

**Results:**

Race/ethnic groups differed in psychosocial correlates of childhood BMI at age 9 and 10. Among non-Latino White children, higher family income (B = −0.086, p < 0.001), higher parental education (B = −0.069, p < 0.001), and living in a married household (B = −0.079, p < 0.001) were associated with lower BMI. Additionally, the presence of healthy food options in the zip code (B = −0.030, p = 0.032) was linked to lower BMI, while lack of planning (B = 0.032, p = 0.030) was associated with higher BMI. For non-Latino Black children, positive urgency (B = −0.068, p = 0.022) was negatively associated with BMI, while other factors such as family SES and neighborhood SES did not show significant associations. For Latino children, higher family income (B = −0.093, p = 0.001) and parental education (B = −0.099, p < 0.001) were associated with lower BMI. In this group, male gender (B = 0.043, p = 0.033) was associated with higher BMI. Among Asian children, higher family income (B = −0.199, p = 0.006) and parental education (B = −0.144, p = 0.037) were significantly associated with lower BMI. For children in the “Other” racial/ethnic category, higher family income (B = −0.101, p = 0.023), living in a married household (B = −0.076, p = 0.026), and higher median income in the zip code (B = −0.083, p = 0.013) were associated with lower BMI. In this group, male children had lower BMI compared to females (B = −0.089, p = 0.001).

**Conclusion:**

The findings highlight substantial racial/ethnic differences in the psychosocial and socioeconomic correlates of BMI in children. There is a need for tailored interventions that target social determinants of childhood high BMI. One size does not fit all.

## 1. Introduction

Body Mass Index (BMI), a widely used indicator of weight relative to height, varies significantly across racial and ethnic groups among children in the United States [1]. These differences often reflect underlying disparities in socioeconomic resources and neighborhood environments that shape differences in individual-level behavioral traits [2]. High BMI is a critical health concern during childhood as it is associated with adverse physical and psychosocial outcomes, making it important to understand the factors contributing to these disparities [3].

Studies consistently show that Black and Latino children have higher BMI compared to their non-Latino White peers, while Asian children tend to have lower BMI [1,4–7]. These patterns suggest that structural and contextual factors contribute to racial and ethnic disparities in BMI [8]. For Black and Latino children, systemic inequities such as reduced access to resources, differences in food environments, and limited recreational opportunities may amplify the risk of higher BMI [9]. Understanding these disparities is essential for addressing the broader health and social inequities that underlie them.

Family socioeconomic status (SES) is a crucial determinant of children’s BMI [10–14]. Higher family SES is generally associated with better access to resources that promote healthy behaviors, such as nutritious food and extracurricular activities. However, research indicates that the protective effects of family SES may be weaker for Black and Latino children than for their non-Latino White peers, a phenomenon referred to as Minorities’ Diminished Returns (MDRs) [15,16]. These differences highlight the need to examine how family SES contributes to BMI across racial and ethnic groups.

Neighborhood SES, reflecting the broader environmental context in which families reside, also plays an important role in shaping children’s BMI [5,17–21]. Living in a high-SES neighborhood often provides access to healthy food options, safe spaces for physical activity, and supportive social networks. However, marginalized groups may experience reduced benefits from such environments due to systemic inequities, such as residential segregation and resource distribution, which can limit opportunities for maintaining a healthy weight.

In addition to socioeconomic factors, individual-level behavioral traits such as inhibitory control are critical psychosocial correlates of BMI [22–29]. Impulsivity and the motivation to pursue immediate gratification, may drive preferences for energy-dense foods, while deficits in inhibitory control can undermine efforts to regulate eating behaviors. These traits, shaped by both genetic and environmental influences, may vary by race and ethnicity, reflecting cultural and contextual differences in food-related behaviors and stress responses.

Two competing hypotheses offer different perspectives on the relationship between socioeconomic and behavioral factors and BMI disparities across racial and ethnic groups. On one hand, racial and ethnic disparities in BMI and other health outcomes can be understood through frameworks such as the multiple jeopardy hypothesis, double disadvantage theory, and cumulative life-course disadvantage. These frameworks propose that adversities disproportionately experienced by Black, Latino, and other marginalized racial/ethnic groups compound over time, amplifying the effects of individual risk factors. For children in these groups, each socioeconomic disadvantage or behavioral risk factor may carry greater weight compared to their non-Latino White peers. This amplification arises from the intersection of multiple adversities, such as poverty, systemic racism, and limited access to health-promoting resources, which interact to exacerbate vulnerability. Over time, these compounded adversities result in cumulative disadvantage, intensifying the impact of low family SES, low neighborhood SES, or behavioral challenges like impulsivity. Consequently, this hypothesis suggests that Black and Latino children will exhibit stronger associations between risk factors and BMI, as these adversities create a feedback loop that magnifies negative outcomes.

Conversely, the minorities’ diminished returns (MDRs) or marginalization-related diminished returns framework offers a different perspective [30,31]. This theory posits that structural barriers limit the relevance and protective effects of SES for marginalized groups, suggesting that higher SES may have less influence on BMI outcomes for Black and Latino children compared to their non-Latino White counterparts. These diminished returns occur because systemic inequities, such as discrimination, neighborhood segregation, and unequal access to resources, constrain the ability of higher SES to fully translate into better health outcomes. According to this hypothesis, socioeconomic and behavioral factors might exhibit weaker associations with BMI for Black and Latino children, while stronger associations are observed for White children who are better positioned to capitalize on SES-related advantages.

Together, these two hypotheses present opposing predictions: the first suggests stronger associations between risk factors and BMI for Black and Latino children due to cumulative disadvantage, while the second implies stronger associations for non-Latino White children due to the full realization of SES advantages in this group. Understanding which hypothesis aligns more closely with observed data is essential for tailoring interventions to reduce disparities effectively.

This study uses data from the Adolescent Brain Cognitive Development (ABCD) study [32–38] to examine racial and ethnic differences in family and neighborhood SES, and inhibitory control as correlates of BMI among 9–10-year-old children. By exploring these factors across non-Latino White, Black, Latino, Asian, and Other racial/ethnic groups, this research aims to uncover the nuanced drivers of disparities in BMI and inform interventions tailored to the diverse needs of U.S. children.

## 2. Methods

### Study Design

This study used cross-sectional data from the Adolescent Brain Cognitive Development (ABCD) study [32–38], collected in 2016. The ABCD study is a nationally representative cohort of children aged 9–10 years in the United States. Participants were recruited from diverse racial/ethnic backgrounds, including non-Latino White, Black, Latino, Asian, and Other racial/ethnic groups. The analysis was limited to children with complete data on BMI, family SES, neighborhood SES, and psychosocial measures (N = [insert number]).

### Analytical sample

#### Measures

##### Outcome Variable (Body Mass Index; BMI):

BMI was calculated using objectively measured weight and height, standardized for age and sex using CDC growth charts.

##### Predictor Variables:

###### Family Socioeconomic Status (SES):

Family SES was measured as a composite variable including parental education (highest level attained by either parent), and household income (categorized into income brackets).

###### Neighborhood SES:

Neighborhood SES was measured using census-tract-level data on income.

###### Impulsivity:

Using the UPPS-P (Urgency, (lack of) Premeditation, (lack of) Perseverance, Sensation Seeking, and Positive Urgency), also called Impulsive Behavior Scale, ABCD assessed positive urgency, negative urgency, lack of planning, and sensation seeking. Positive urgency refers to the tendency to act impulsively in response to extreme positive emotions. Negative urgency reflects the tendency to act impulsively during intense negative emotions. Lack of planning (also called lack of premeditation) captures difficulty in thinking through the consequences of actions before acting. Sensation seeking represents the tendency to seek out novel and thrilling experiences. These components of impulsivity reflect children’s ability to delay gratification and regulate impulsive behaviors. The UPPS-P is widely used to assess distinct dimensions of impulsivity. All measures are continuous, with higher scores indicating greater impulsivity across domains.

##### Covariates:

Covariates included child age, sex. Sex was 1 for male and 0 for female. Age was 9 or 10 year old.

##### Moderators (Stratifying variables):

Racial/ethnic categories were self-reported as non-Latino White (reference category), Black, Latino, Asian, or Other.

### Statistical Analysis

Descriptive statistics for BMI were calculated by race/ethnicity. A multivariable structural equation model (SEM) examined the associations between family SES, neighborhood SES, and inhibitory control with BMI in the overall sample. Next, multi-group SEMs were conducted, stratified by race/ethnicity, to assess differences in the strength and direction of these associations. All analyses were performed using Stata/SE 16.0, with statistical significance set at p < 0.05.

### Ethics Approval

The ABCD study received institutional review board (IRB) approval from the University of California San Diego (UCSD) and all participating research sites. Informed consent was obtained from adult participants, and assent was provided by child participants. This secondary analysis was deemed exempt by the IRB at Charles R. Drew University of Medicine and Science [IRB Net = 1811119–1].

### Data Availability

The ABCD study data are available for researchers, using an open science model, through the National Institute of Mental Health (NIMH) Data Archive, accessible via NDA website.

## 3. Results

As Table 1 shows, BMI was, on average, higher for Black and Latino children compared to non-Latino White and Asian children. Specifically, the mean BMI was 20.58 (SE = 0.13, 95% CI: 20.33–20.83) for Black children and 20.03 (SE = 0.09, 95% CI: 19.85–20.21) for Latino children. In contrast, non-Latino White children had a mean BMI of 17.90 (SE = 0.04, 95% CI: 17.81–17.98), and Asian children had the lowest mean BMI at 17.74 (SE = 0.25, 95% CI: 17.26–18.22). Children categorized as “Other” fell in between these groups, with a mean BMI of 18.83 (SE = 0.12, 95% CI: 18.59–19.06). These findings highlight notable racial and ethnic differences in BMI among 9–10-year-old children.

Table 2 presents the bivariate correlations between BMI and various demographic, socioeconomic, neighborhood, and behavioral variables in the overall sample. Socioeconomic variables were negatively correlated with BMI. Family income (r = −0.26, p < 0.05), parental education (r = −0.23, p < 0.05), married household status (r = −0.19, p < 0.05), and zip code median income (r = −0.18, p < 0.05) were all inversely associated with BMI, indicating that higher SES was linked to lower BMI. Access to healthy food in the zip code was also negatively correlated with BMI, though the relationship was weaker (r = −0.12, p < 0.05). Positive urgency was positively correlated with BMI (r = 0.04, p < 0.05), while sensation seeking was negatively correlated (r = −0.03, p < 0.05). Negative urgency and lack of planning were not significantly correlated with BMI. Age showed a small positive correlation with BMI (r = 0.07, p < 0.05), while male gender was negatively correlated with BMI (r = −0.03, p < 0.05).

As shown by Table 3, in the overall sample, family income (B = −0.120, p < 0.001), parental education (B = −0.100, p < 0.001), living in a married household (B = −0.070, p < 0.001), and higher median income in the zip code (B = −0.061, p < 0.001) were all inversely associated with BMI, indicating that higher family and neighborhood SES were protective factors against higher BMI. Behavioral traits, such as UPPS positive urgency (B = −0.020, p = 0.052), approached significance but did not meet the threshold, while other traits like lack of planning, sensation seeking, and negative urgency showed no significant associations with BMI. Healthy food availability in the zip code was also not significantly associated with BMI in the overall sample (B = −0.007, p = 0.556). Older age was linked to higher BMI (B = 0.080, p < 0.001), while male children had slightly lower BMI compared to females (B = −0.023, p = 0.011).

As Table 4 shows, the associations between psychosocial and socioeconomic factors and BMI varied across racial and ethnic groups. Among non-Latino White children, higher family income (B = −0.086, p < 0.001), higher parental education (B = −0.069, p < 0.001), and living in a married household (B = −0.079, p < 0.001) were associated with lower BMI. Additionally, the presence of healthy food options in the zip code (B = −0.030, p = 0.032) was linked to lower BMI, while lack of planning (B = 0.032, p = 0.030) was associated with higher BMI.

For non-Latino Black children, positive urgency (B = −0.068, p = 0.022) was negatively associated with BMI, while other factors such as family SES and neighborhood SES did not show significant associations.

For Latino children, higher family income (B = −0.093, p = 0.001) and parental education (B = −0.099, p < 0.001) were associated with lower BMI. In this group, male gender (B = 0.043, p = 0.033) was associated with higher BMI.

Among Asian children, higher family income (B = −0.199, p = 0.006) and parental education (B = −0.144, p = 0.037) were significantly associated with lower BMI.

For children in the “Other” racial/ethnic category, higher family income (B = −0.101, p = 0.023), living in a married household (B = −0.076, p = 0.026), and higher median income in the zip code (B = −0.083, p = 0.013) were associated with lower BMI. In this group, male children had lower BMI compared to females (B = −0.089, p = 0.001).

## 4. Discussion

This study aimed to explore the associations of family socioeconomic status (SES), neighborhood SES, and inhibitory control with body mass index (BMI) among 9–10-year-old children overall and by race/ethnicity. Using data from the Adolescent Brain Cognitive Development (ABCD) study, we sought to examine whether these relationships vary by race and ethnicity, focusing on differences between non-Latino White, Black, Latino, Asian, and Other racial/ethnic groups. By identifying these associations, the study aimed to shed light on the interplay of socioeconomic and behavioral factors in shaping BMI and to better understand the disproportionate burden of higher BMI observed among Black and Latino children.

In the overall sample, family income (B = −0.120, p < 0.001), parental education (B = −0.100, p < 0.001), living in a married household (B = −0.070, p < 0.001), and higher median income in the zip code (B = −0.061, p < 0.001) were all inversely associated with BMI, indicating that higher family and neighborhood SES were protective factors against higher BMI. Behavioral traits, such as UPPS positive urgency (B = −0.020, p = 0.052), approached significance but did not meet the threshold, while other traits like lack of planning, sensation seeking, and negative urgency showed no significant associations with BMI. Healthy food availability in the zip code was also not significantly associated with BMI in the overall sample (B = −0.007, p = 0.556). Older age was linked to higher BMI (B = 0.080, p < 0.001), while male children had slightly lower BMI compared to females (B = −0.023, p = 0.011).

Race/ethnic groups differed in psychosocial correlates of childhood BMI at age 9 and 10. Among non-Latino White children, higher family income (B = −0.086, p < 0.001), higher parental education (B = −0.069, p < 0.001), and living in a married household (B = −0.079, p < 0.001) were associated with lower BMI. Additionally, the presence of healthy food options in the zip code (B = −0.030, p = 0.032) was linked to lower BMI for White individuals but not other groups. In addition, lack of planning (B = 0.032, p = 0.030) was associated with higher BMI for non-Latino White participants only. For non-Latino Black children, positive urgency (B = −0.068, p = 0.022) was negatively associated with BMI, while other factors such as family SES and neighborhood SES did not show significant associations. For Latino children, higher family income (B = −0.093, p = 0.001) and parental education (B = −0.099, p < 0.001) were associated with lower BMI. In this group, male gender (B = 0.043, p = 0.033) was associated with higher BMI. Among Asian children, higher family income (B = −0.199, p = 0.006) and parental education (B = −0.144, p = 0.037) were significantly associated with lower BMI. For children in the “Other” racial/ethnic category, higher family income (B = −0.101, p = 0.023), living in a married household (B = −0.076, p = 0.026), and higher median income in the zip code (B = −0.083, p = 0.013) were associated with lower BMI. In this group, male children had lower BMI compared to females (B = −0.089, p = 0.001).

Existing literature underscores the importance of family and neighborhood SES in determining children’s health outcomes, including BMI. Family SES is associated with access to resources such as healthier food, healthcare, and extracurricular opportunities that promote physical activity, while neighborhood SES is linked to environmental characteristics such as the availability of safe play areas, proximity to grocery stores with nutritious food options, and community-level stressors [39,40]. Inhibitory control, a key component of self-regulation, is critical for resisting high-calorie, low-nutrition foods. Together, these factors are thought to interact in complex ways to influence BMI, though how these dynamics differ by race and ethnicity remains underexplored.

Family SES provides a foundation for children’s development by shaping their daily environments, access to healthy food, educational opportunities, and extracurricular activities. Higher family SES is generally associated with lower BMI due to greater exposure to health-promoting resources. Neighborhood SES, on the other hand, reflects the broader community context in which children grow up. Living in a high-SES neighborhood typically provides better access to supermarkets offering healthy food, safer streets for physical activity, and reduced exposure to chronic stressors such as violence or pollution. However, marginalized groups may face systemic barriers that limit the benefits of these environments, such as under-resourced schools or racial discrimination, which may exacerbate disparities in BMI.

In line with our previous research documenting race and ethnic differences in the psychosocial correlates of BMI [41–50], we observed substantial race and ethnic variation in the effects of multiple psychosocial factors as risk or protective factors for childhood BMI. Specifically, our findings highlight differential roles of socioeconomic status (SES), inhibitory control, impulsivity, neighborhood food availability, and even demographic factors such as age and gender. These variations suggest that the same psychosocial factors do not operate uniformly across racial and ethnic groups, reinforcing the importance of context in shaping childhood obesity risk. Our results underscore the necessity of applying an intersectionality framework [51–55] to better understand and address the complex and multidimensional psychosocial determinants of childhood obesity in the U.S. Such an approach can help tailor interventions that account for the unique social, economic, and behavioral contexts shaping obesity risk among diverse populations.

This study is cross-sectional in design, which limits its ability to establish causality or directionality in the observed relationships. Although we used well-validated measures for SES and behavioral traits, some constructs, such as neighborhood SES, relied on proxy indicators and may not fully capture the lived experiences of participants. Additionally, unmeasured factors such as diet, physical activity, or cultural practices were not included in the analysis and may have influenced the observed associations. Finally, while the sample was diverse, some racial/ethnic groups, particularly Asian and Other categories, were underrepresented, limiting generalizability.

Future studies should use longitudinal designs to examine how the relationships between SES, behavioral traits, and BMI evolve over time and across developmental stages. Research should also explore the role of cultural, structural, and contextual factors in shaping these associations, particularly among Black and Latino children. Interventions targeting both socioeconomic and behavioral factors should be tested to determine their effectiveness in reducing BMI disparities. Additionally, future work should consider qualitative approaches to better understand the lived experiences of families and neighborhoods and their influence on BMI.

These new findings underscore the critical need to address both family and neighborhood SES in public health interventions aimed at reducing BMI disparities in children. Policies that promote equitable access to resources, such as affordable housing in high-SES neighborhoods, quality education, and community-based programs, are essential for addressing these disparities. Behavioral interventions targeting inhibitory control may not be similarly beneficial for diverse children who experience disproportionate challenges in managing BMI. By addressing the broader structural and systemic barriers that underlie these disparities, health equity for all children can be promoted.

## 5. Conclusions

This study highlights the significant racial and ethnic differences in the socioeconomic and behavioral correlates of BMI in children. Our findings emphasize the need for tailored, equity-focused interventions to address the root causes of BMI inequality and promote healthier outcomes for all children. Promoting health equity requires a multifaceted approach that addresses both the individual and structural determinants of BMI.

## Figures and Tables

**Figure 1.**
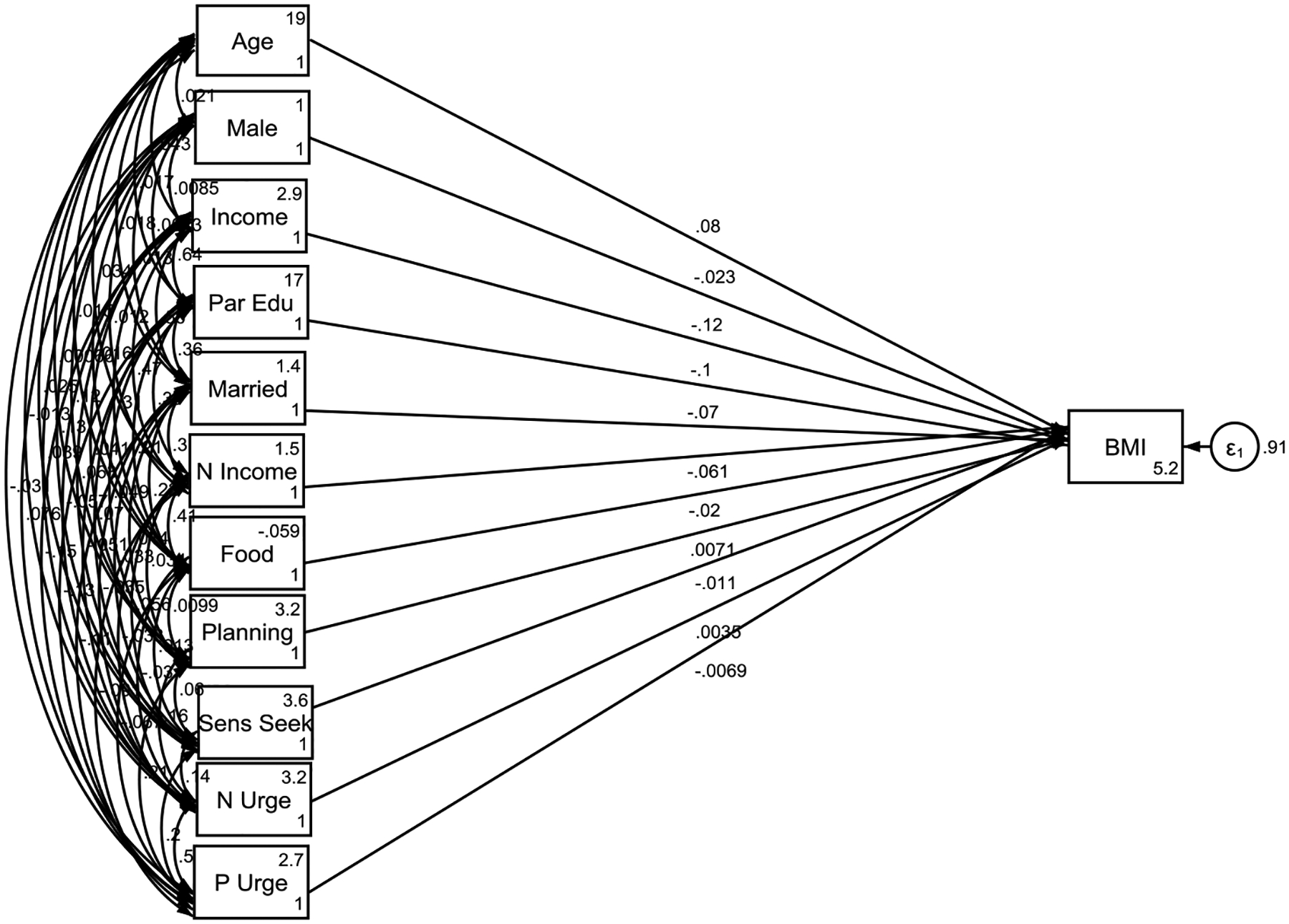
Psychosocial correlates of childhood body mass index Overall **Note:** P Urge = Positive Urgency; **N Urge** = Negative Urgency; Sens Seek= Sensation Seeking; Planning= Lack of Planning; Par Edu= Parental Education; N Income= Neighborhood Income; Married= Married Household; Food= Healthy Food Availability in the Zip Code.

**Figure 2.**
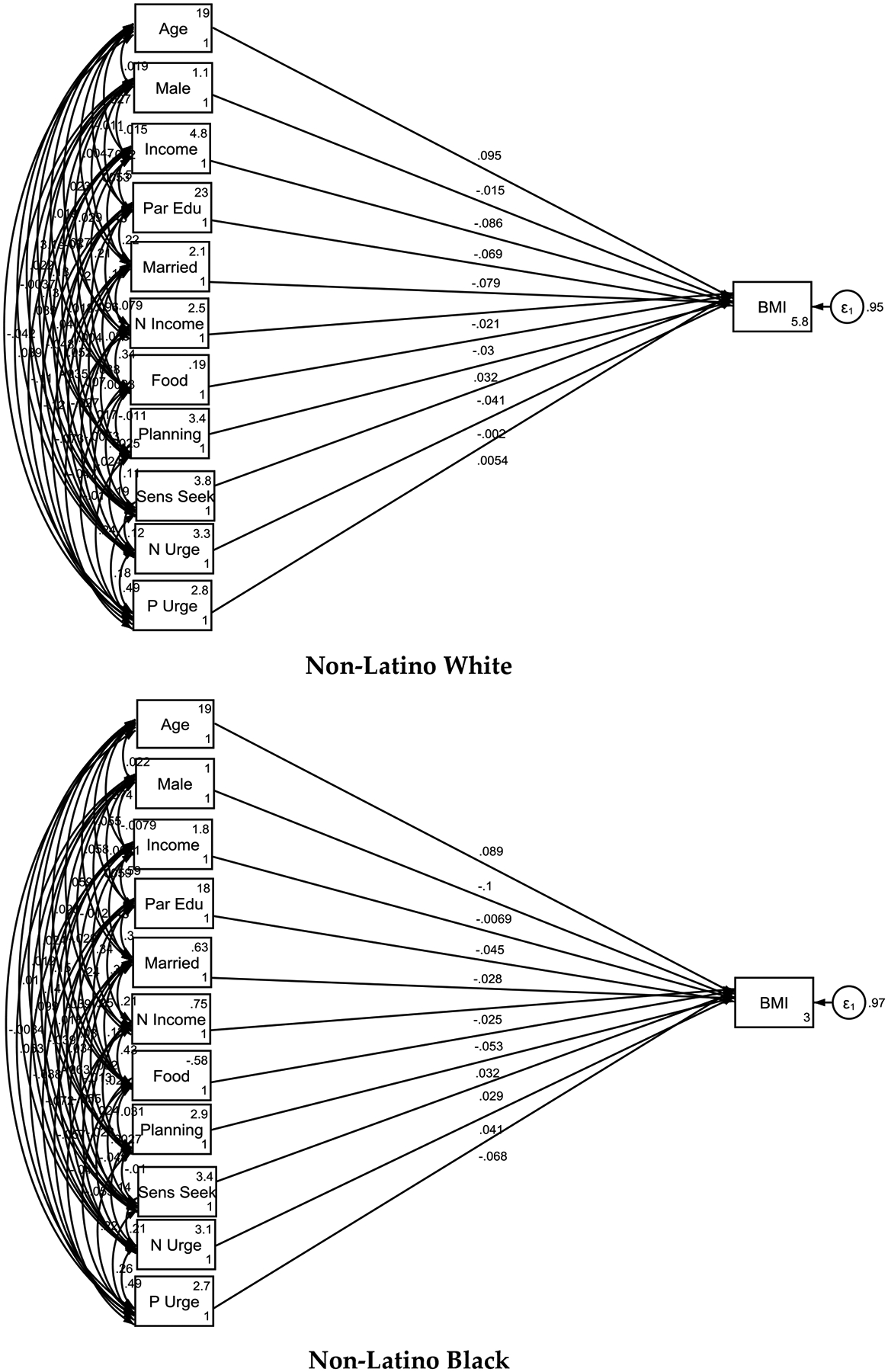

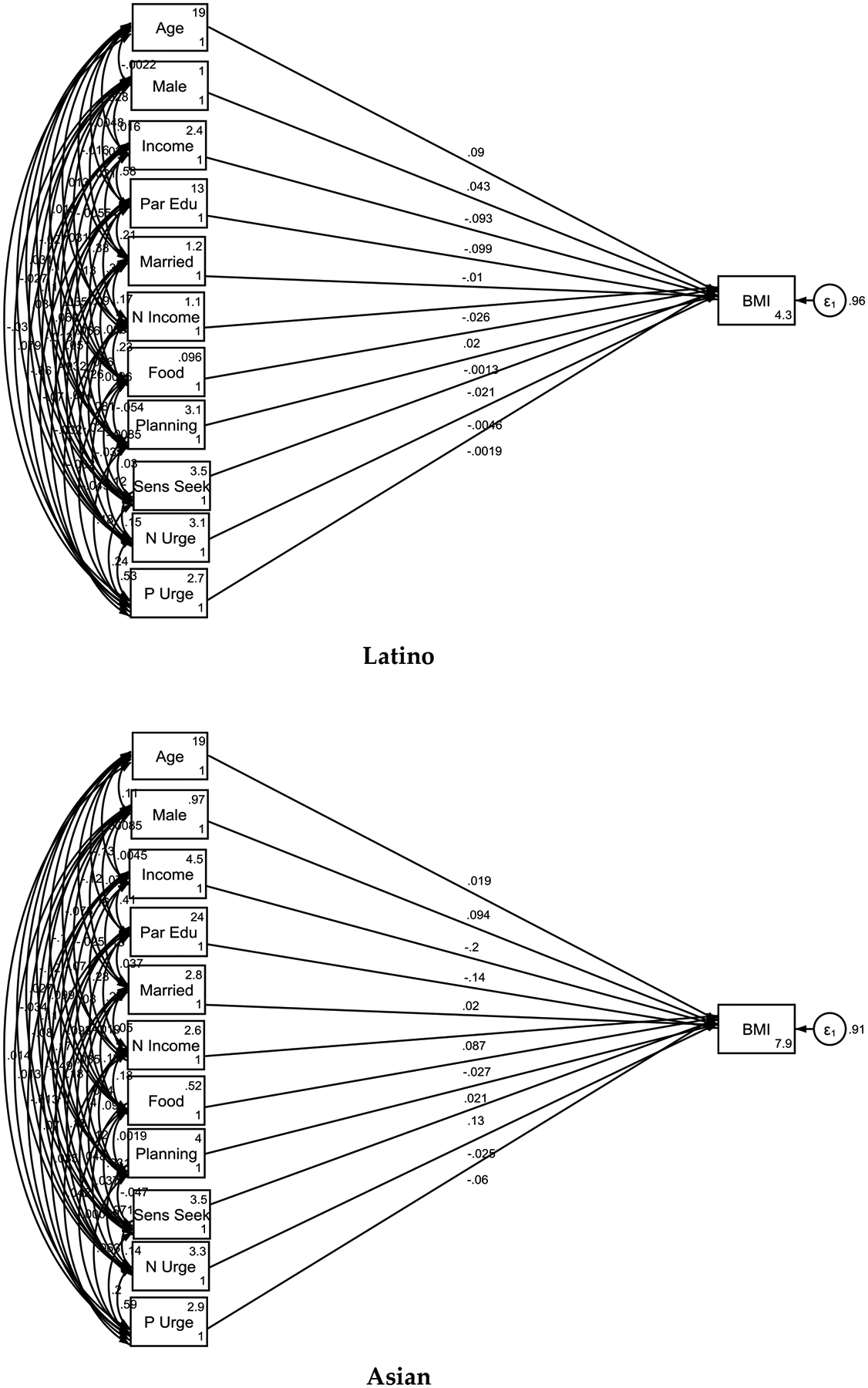

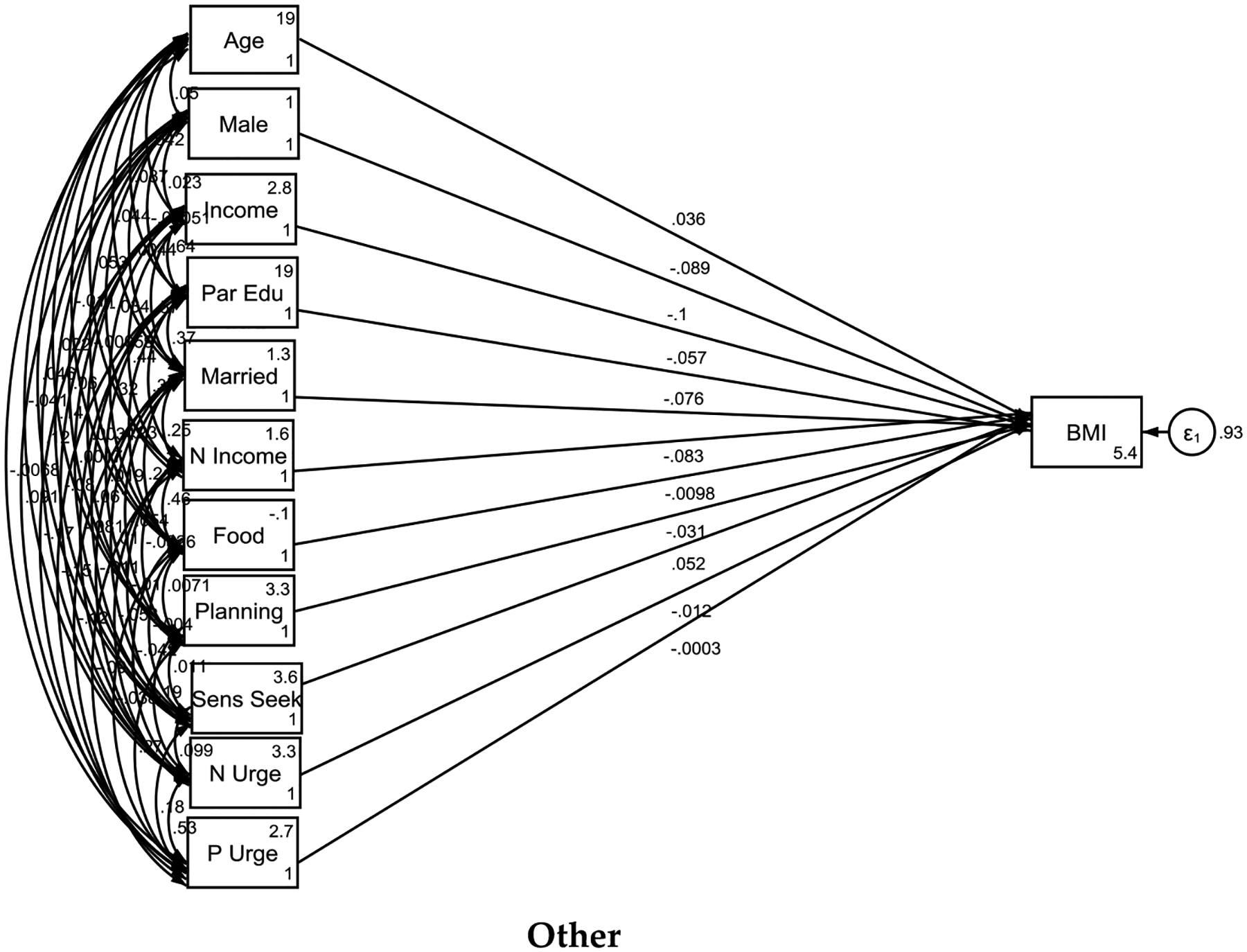
Psychosocial correlates of childhood body mass index by race/Ethnicity **Note:** P Urge = Positive Urgency; **N Urge** = Negative Urgency; Sens Seek= Sensation Seeking; Planning= Lack of Planning; Par Edu= Parental Education; N Income= Neighborhood Income; Married= Married Household; Food= Healthy Food Availability in the Zip Code.

**Table 1.** Mean Body Mass Index by Race/Ethnicity

	Mean	SE	95%	CI
Non-Latino White	17.90	0.04	17.81	17.98
Non-Latino Black	20.58	0.13	20.33	20.83
Latino	20.03	0.09	19.85	20.21
Asian	17.74	0.25	17.26	18.22
Other	18.83	0.12	18.59	19.06

**Table 2.** Bivariate Correlations, Overall

	1	2	3	4	5	6	7	8	9	10	11	12
1 Body Mass Index (BMI)	1.00											
2 Age	0.07*	1.00										
3 Male	−0.03*	0.02	1.00									
4 Family Income	−0.26*	0.04*	0.01	1.00								
5 Parental Education	−0.23*	0.02	0.00	0.62*	1.00							
6 Married Household	−0.19*	0.02	0.01	0.55*	0.36*	1.00						
7 Zip Code Median Income	−0.18*	0.03*	0.01	0.46*	0.39*	0.30*	1.00					
8 Zip Code Healthy Food	−0.12*	0.01	0.01	0.31*	0.21*	0.23*	0.40*	1.00				
9 UPPS Positive Urgency	0.04*	−0.03*	0.08*	−0.14*	−0.13*	−0.11*	−0.09*	−0.07*	1.00			
10 UPPS Negative Urgency	0.01	−0.01	0.09*	−0.06*	−0.05*	−0.04*	−0.03*	−0.03*	0.50*	1.00		
11 UPPS Lack of Planning	−0.01	0.00	0.12*	0.04*	0.05*	0.02	0.03*	0.01	0.21*	0.16*	1.00	
12 UPPS Sensation Seeking	−0.03*	0.02*	0.13*	0.06*	0.07*	0.03*	0.06*	0.01	0.20*	0.14*	0.06*	1.00

Note:

* p<0.05

UPPS: Urgency, (lack of) Premeditation, (lack of) Perseverance, Sensation Seeking, and Positive Urgency

**Table 3.** Psychosocial determinations of childhood baseline body mass index at baseline, Overall

Independent Variable		Dependent Variable	Beta	SE	95%	CI	p
Age (Years)	->	BMI	0.080	0.009	0.062	0.097	< 0.001
Gender (Male)	->	BMI	−0.023	0.009	−0.040	−0.005	0.011
Family Income (1–10)	->	BMI	−0.120	0.014	−0.148	−0.092	< 0.001
Parental Education	->	BMI	−0.100	0.012	−0.123	−0.077	< 0.001
Married Household	->	BMI	−0.070	0.011	−0.091	−0.049	< 0.001
Median Income in Zip Code	->	BMI	−0.061	0.011	−0.082	−0.040	< 0.001
Healthy Food in Zip Code	->	BMI	−0.007	0.012	−0.030	0.016	0.556
UPPS Positive Urgency	->	BMI	−0.020	0.010	−0.040	0.000	0.052
UPPS Lack of Planning	->	BMI	0.007	0.010	−0.013	0.027	0.479
UPPS Sensation Seeking	->	BMI	−0.011	0.010	−0.031	0.009	0.269
UPPS Negative Urgency	->	BMI	0.004	0.011	−0.019	0.026	0.755
Intercept	->	BMI	5.227	0.250	4.737	5.717	< 0.001

Note: UPPS: Urgency, (lack of) Premeditation, (lack of) Perseverance, Sensation Seeking, and Positive Urgency

**Table 4.** Psychosocial determinations of childhood baseline body mass index at baseline, by race/ethnicity

Independent Variable		Dependent Variable	B	SE	[95%	CI]	P
**Non-Latino White**							
Age (Years)	->	BMI	0.095	0.012	0.070	0.119	< 0.001
Gender (Male)	->	BMI	−0.015	0.013	−0.039	0.010	0.254
Family Income (1–10)	->	BMI	−0.086	0.017	−0.119	−0.054	0.000
Parental Education	->	BMI	−0.069	0.015	−0.098	−0.041	< 0.001
Married Household	->	BMI	−0.079	0.014	−0.106	−0.051	< 0.001
Median Income in Zip Code	->	BMI	−0.021	0.014	−0.047	0.006	0.124
Healthy Food in Zip Code	->	BMI	−0.030	0.014	−0.057	−0.003	0.032
UPPS Positive Urgency	->	BMI	0.005	0.017	−0.028	0.038	0.746
UPPS Lack of Planning	->	BMI	0.032	0.015	0.003	0.061	0.030
UPPS Sensation Seeking	->	BMI	−0.041	0.015	−0.070	−0.012	0.005
UPPS Negative Urgency	->	BMI	−0.002	0.016	−0.034	0.030	0.902
Intercept	->	BMI	5.763	0.399	4.980	6.545	< 0.001
							
**Non-Latino Black**							
Age (Years)	->	BMI	0.089	0.023	0.043	0.135	< 0.001
Gender (Male)	->	BMI	−0.102	0.024	−0.149	−0.055	< 0.001
Family Income (1–10)	->	BMI	−0.007	0.035	−0.075	0.061	0.842
Parental Education	->	BMI	−0.045	0.030	−0.104	0.015	0.140
Married Household	->	BMI	−0.028	0.027	−0.080	0.024	0.290
Median Income in Zip Code	->	BMI	−0.025	0.027	−0.079	0.029	0.360
Healthy Food in Zip Code	->	BMI	−0.053	0.028	−0.109	0.002	0.060
UPPS Positive Urgency	->	BMI	−0.068	0.030	−0.125	−0.010	0.022
UPPS Lack of Planning	->	BMI	0.032	0.026	−0.019	0.082	0.224
UPPS Sensation Seeking	->	BMI	0.029	0.026	−0.023	0.080	0.275
UPPS Negative Urgency	->	BMI	0.041	0.029	−0.015	0.098	0.150
Intercept	->	BMI	2.992	0.692	1.637	4.348	< 0.001
							
**Latino**							
Age (Years)	->	BMI	0.090	0.020	0.051	0.129	< 0.001
Gender (Male)	->	BMI	0.043	0.020	0.004	0.083	0.033
Family Income (1–10)	->	BMI	−0.093	0.029	−0.149	−0.037	0.001
Parental Education	->	BMI	−0.099	0.025	−0.149	−0.050	< 0.001
Married Household	->	BMI	−0.010	0.022	−0.053	0.033	0.641
Median Income in Zip Code	->	BMI	−0.026	0.023	−0.070	0.018	0.252
Healthy Food in Zip Code	->	BMI	0.020	0.022	−0.023	0.063	0.355
UPPS Positive Urgency	->	BMI	−0.002	0.027	−0.055	0.051	0.945
UPPS Lack of Planning	->	BMI	−0.001	0.023	−0.046	0.043	0.955
UPPS Sensation Seeking	->	BMI	−0.021	0.023	−0.066	0.025	0.375
UPPS Negative Urgency	->	BMI	−0.005	0.026	−0.056	0.047	0.862
Intercept	->	BMI	4.346	0.506	3.355	5.338	< 0.001
							
**Asian**							
Age (Years)	->	BMI	0.019	0.063	−0.104	0.142	0.763
Gender (Male)	->	BMI	0.094	0.064	−0.030	0.219	0.138
Family Income (1–10)	->	BMI	−0.199	0.073	−0.341	−0.056	0.006
Parental Education	->	BMI	−0.144	0.069	−0.279	−0.009	0.037
Married Household	->	BMI	0.020	0.066	−0.108	0.149	0.756
Median Income in Zip Code	->	BMI	0.087	0.065	−0.040	0.215	0.180
Healthy Food in Zip Code	->	BMI	−0.027	0.065	−0.155	0.100	0.676
UPPS Positive Urgency	->	BMI	−0.060	0.081	−0.219	0.099	0.457
UPPS Lack of Planning	->	BMI	0.021	0.066	−0.109	0.151	0.751
UPPS Sensation Seeking	->	BMI	0.127	0.068	−0.006	0.260	0.061
UPPS Negative Urgency	->	BMI	−0.025	0.083	−0.187	0.137	0.759
Intercept	->	BMI	7.928	2.091	3.830	12.027	< 0.001
							
**Other**							
Age (Years)	->	BMI	0.036	0.028	−0.018	0.090	0.186
Gender (Male)	->	BMI	−0.089	0.028	−0.144	−0.034	0.001
Family Income (1–10)	->	BMI	−0.101	0.044	−0.187	−0.014	0.023
Parental Education	->	BMI	−0.057	0.036	−0.128	0.015	0.121
Married Household	->	BMI	−0.076	0.034	−0.142	−0.009	0.026
Median Income in Zip Code	->	BMI	−0.083	0.033	−0.148	−0.017	0.013
Healthy Food in Zip Code	->	BMI	−0.010	0.033	−0.074	0.054	0.764
UPPS Positive Urgency	->	BMI	0.000	0.038	−0.074	0.074	0.994
UPPS Lack of Planning	->	BMI	−0.031	0.032	−0.093	0.032	0.336
UPPS Sensation Seeking	->	BMI	0.052	0.031	−0.010	0.113	0.099
UPPS Negative Urgency	->	BMI	−0.012	0.036	−0.083	0.059	0.745
Intercept	->	BMI	5.401	0.808	3.817	6.985	< 0.001

Note: BMI: Body Mass Index; UPPS: Urgency, (lack of) Premeditation, (lack of) Perseverance, Sensation Seeking, and Positive Urgency
